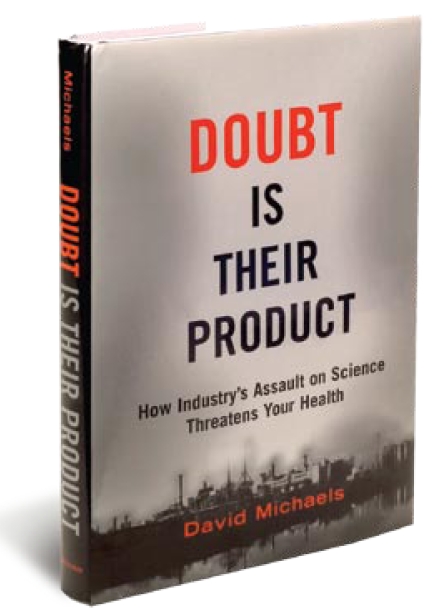# Doubt Is Their Product: How Industry’s Assault on Science Threatens Your Health

**Published:** 2009-05

**Authors:** Steven G. Gilbert

**Affiliations:** Steven G. Gilbert is director and founder of the Institute of Neurotoxicology and Neurological Disorders, Seattle, Washington, and recently started Toxipedia (www.toxipedia.org). He writes and lectures on issues related to toxicology, drug development, and bioethics

*By David Michaels* New York: Oxford University Press, 2008. 327 pp. ISBN: 978-0-19-530067-3, $27.95

If your time is limited and you are looking for a plan of action to improve the courts and public health regulatory system, start with the last two chapters of David Michaels’ *Doubt Is Their Product*, which provide steps we can take today to move occupational and environmental health policy forward. Then go back and read the entire book.

*Doubt Is Their Product* presents the ways business interests take advantage of scientific and regulatory processes to obscure the need to address the many current global occupational and environmental problems. Part of the scientific process is to explore and describe the uncertainty that is part of every scientific endeavor. The very nature of scientific exploration is to ask and answer the next question. But rather than accepting the process of scientific discovery, business interests press to have every tiny bit of uncertainty explored before any policy decision can be made, demanding proof rather than precaution—in fact, they even manufacture uncertainty. As a result, decisions are not made; policy is not advanced; problems are not addressed.

The historical illustrations Michaels uses are as compelling as they are frustrating: Why must so much illness and death occur before action is taken? Michaels points out that the health consequences of asbestos exposure were first recognized in the late 1800s. Also, shortly after the development of synthetic dyes in the mid 1800s, it was soon apparent that dye workers developed bladder cancer. The health consequences of lead exposure have been recognized for > 2,000 years. Although Europe banned lead-based paint in the 1920s, the United States, despite the scientific evidence, did not finally ban the use of lead-based paints until 1978. It was not the scientific evidence that was lacking, but rather the public and political will. For multiple cases, Michaels documents how businesses made it their business to confuse and mislead the public and decision makers. He provides a fascinating account of some of the people and consulting groups that businesses funded specifically to delay or confuse the decision-making process.

Michaels describes the efforts of the tobacco industry to obscure the scientific evidence of the adverse health effects of their products while increasing sales by manipulating nicotine levels and by advertising to young people. (The book’s title is derived from a tobacco industry memo describing how doubt can be used to counter fact.) The consequences and ultimate cost of those adverse health effects, such as cancer and cardiovascular disease, are borne by individuals and the general public. The tobacco industry has spent millions to emphasize the uncertainty around scientific findings with the goal of slowing recognition that tobacco use has been a public health disaster.

Michaels brings an insider’s perspective and also provides extensive references to support his position. As Assistant Secretary of Energy for the Environment, Safety, and Health in the Department of Energy during the Clinton administration, he worked to acknowledge exposures related to the nuclear weapons industry and consequent health-related issues for workers. He faced the challenge of developing a fair compensation system that recognized the workers who sacrifice their health for their jobs and national security. These experiences provide the passion and motivation for this book.

An underlying theme of this book is that capitalism, as currently practiced, prioritizes short-term profits and increased return to investors over human and environmental health. Businesses are driven by two primary priorities: to increase revenue by building and selling more products, and to maximize profits by externalizing costs to another entity. Often this other entity is the public or workers. The public pays for the costs of illness and pollution incurred by workers and the environment; immediate business profits are often valued more highly than the health of workers, public, or the environment.

Michaels points out that when industry pushes for more scientific study, in the face of even slight uncertainty, the promotion of public and environmental health is often impeded. One way to address this is through greater transparency with regard to legal decisions, settlements, and basic information on the health effects of chemicals and other hazards. Another is by greater transparency of the source of funding for scientific research and authors’ conflict of interest.

Better use of the precautionary principle, the acceptance of sufficient but not absolute proof, is another way to accelerate decision making in protecting human and environmental health. *Doubt Is Their Product* is a powerful argument for the need to reinforce our ethical responsibilities to protect human and environmental health even when it requires regulation and increased costs. After all, it would seem only fair for those who profit to acknowledge and be accountable for the true costs of their products.

## Figures and Tables

**Figure:**